# New hybrid conjugate gradient methods with the generalized Wolfe line search

**DOI:** 10.1186/s40064-016-2522-9

**Published:** 2016-06-24

**Authors:** Xiao Xu, Fan-yu Kong

**Affiliations:** College of science, Nanjing University of Science and Technology, Nanjing, 210094 Jiangsu China

**Keywords:** Conjugate gradient, Unconstrained optimization, Line search, Descent property, Global convergence

## Abstract

The conjugate gradient method was an efficient technique for solving the unconstrained optimization problem. In this paper, we made a linear combination with parameters *β*_*k*_ of the DY method and the HS method, and putted forward the hybrid method of DY and HS. We also proposed the hybrid of FR and PRP by the same mean. Additionally, to present the two hybrid methods, we promoted the Wolfe line search respectively to compute the step size *α*_*k*_ of the two hybrid methods. With the new Wolfe line search, the two hybrid methods had descent property and global convergence property of the two hybrid methods that can also be proved.

## Background

### The conjugate gradient method

The conjugate gradient method is a very important and efficient technique for solving large scale minimization problems, due to it can complete with lower storage and simple computation (Birgin and Martinez 2001).

Consider an unconstrained minimization problem.

1 $${\text{min }} f\left( x \right) , \quad { }\,x \in R^{n}$$

where *R*^*n*^ denotes an n-dimensional Euclidean space and $f:R^{n} \mathop{\longrightarrow}\limits{{}}R^{1}$ is a continuously differentiable function. We denote its gradient ∇*f*(*x*_*k*_) by*g*_*k*_. We are concerned with the conjugate gradient methods for solving (1). The iterative process of the conjugate gradient method is given by.

2 $$x_{{{\text{k}} + 1}} = x_{\text{k}} + \alpha_{\text{k}} {\text{d}}_{\text{k}} , \quad {\text{k}} = 1,2, \ldots$$

where *x*_1_ is the initial point and *x*_*k*_ ∊ *R*^*n*^ is the k-th approximation to a solution, *α*_*k*_ is a positive step size, and *d*_*k*_ ∊ *R*^*n*^ is a search direction defined by the following:

3 $$d_{k} = \left\{ \begin{array}{ll} - g_{k},& \quad k = 1 \\ - g_{k} + \beta_{k} d_{k - 1}, &\quad k \ge 2 \\ \end{array} \right.$$

where *β*_*k*_ is a parameter.

### Selection of step size

For selection of step size *α*_*k*_ in the iteration formula, it is generally determined by the direct method (0.618 method), the analytical method (successive difference approximation method), exact line search and inexact line search methods and so on. What we usually used in nonlinear conjugate gradient method is the exact line search and inexact line search. In this paper, we mainly use the Wolfe line search as following

Given $\mu \in \left( {0,\frac{1}{2}} \right)$, $\sigma \in (\mu ,1)$ and *α*_*k*_ > 0 which satisfies

4 $$f(x_{k} ) - f(x_{k} + \alpha_{k} d_{k} ) \ge - \mu \alpha_{k} \nabla f(x_{k} )^{{^{\rm T} }} d_{k}$$

5 $$f(x_{k} ) - f(x_{k} + \alpha_{k} d_{k} ) \ge - \sigma \alpha_{k} \nabla f(x_{k} )^{{^{\rm T} }} d_{k}$$

Generally, the smaller the value of *σ*, the line search will be more precise, but the greater the amount of calculation. So we set *μ* = 0.1, *σ* ∊ [0.6, 0.8]. In order to facilitate analysis, the inexact line search should convert to the exact line search, but in the formula (5), when *σ* → 0, the search is not the exact line search. For this purpose, Fletcher proposed the stronger condition to take the place of (5):

6 $$\left| {g(x_{k} + \alpha_{k} d_{k} )^{\rm T} d_{k} } \right| \le \sigma \left| {g_{k}^{\rm T} d_{k} } \right|$$

We note (4) and (6) as the strong Wolfe line search.

### Selection of search direction

The search direction *d*_*k*_ is generally required to satisfy

7 $${\text{d}}_{\text{k}}^{\text{T}} {\text{g}}_{\text{k}} < 0$$

which guarantees that *d*_*k*_ is a descent direction of *f*(*x*) at *x*_*k*_ (Yuan 1993). In order to maintain the global convergence property, we sometimes require *d*_*k*_ to satisfy a sufficient descent condition

$$g_{k}^{T} d_{k} \le - c\left\| {g_{k} } \right\|^{2}$$

where *c* > 0 is a constant.

### Selection of the parameter *β*_*k*_

Different parameters *β*_*k*_ of different conjugate gradient methods as follows (see (i) Fletcher and Reeves 1964, (ii) Polyak 1969; Polak et al. 1968, (iii) Hestenes and Stiefel 1952, (iv) Vincent 1983, (v), (vi) Dai and Yuan 2000).

$$\begin{aligned} &({\text{i}})\;\beta_{k}^{FR} = \frac{{||g_{k} ||^{2} }}{{||g_{k - 1} ||^{2} }},\quad &({\text{ii}})\;\beta_{k}^{PRP} = \frac{{g_{k}^{T} y_{k - 1} }}{{||g_{k - 1} ||^{2} }},\quad &({\text{iii}})\;\beta_{k}^{HS} = \frac{{g_{k}^{T} y_{k - 1} }}{{d_{k - 1}^{T} y_{k - 1} }} \\ &({\text{iv}})\;\beta _{k}^{{CD}} = - \frac{{||g_{k} ||^{2} }}{{d_{{k - 1}}^{T} g_{{k - 1}} }} ,\quad &({\text{v}})\;\beta_{k}^{LS} = \frac{{g_{k}^{T} y_{k - 1} }}{{ - d_{k - 1}^{T} g_{k - 1} }},\quad &({\text{vi}})\;\beta_{k}^{DY} = \frac{{||g_{k} ||^{2} }}{{d_{k - 1}^{T} y_{k - 1} }} \\ \end{aligned}$$

For positive quadratic function, if we adopt the exact line search, several conjugate gradient methods in the above are equivalent, which implies that the conjugate gradient directions generated by several methods are equivalent. In practical application, the FR method and PRP method are the most common methods.

The FR method is the earliest nonlinear conjugate gradient method, under the exact line search, Powell (1977) pointed out the FR method could continuously produce small steps and have the global efficiency property. Zoutendijk (1970) proved the FR method are always convergence for general non-convex functions; Under the inexact line search, Al-Baali (1985); Liu et al. (1995) proved when $\sigma \le \frac{1}{2}$, the FR method has the global convergence property with strong Wolfe line search. But the shortcoming is the FR method has slow convergence speed.

The PRP method is considered to be the best conjugate gradient method in numerical experiment results at present. Once produce a small step size, the next search direction generated by the PRP algorithm will approach the negative gradient direction automatically. It’s good to avoid the shortcoming that the FR method could continuously produce small steps. Under the exact line search, the PRP method has global convergence property for uniformly convex functions, but it is not established for general non-convex functions (Powell 1984). Under the inexact line search, if search direction is descent direction and the objective function is uniformly convex function, Yuan (1995) proved the PRP method has the global convergence property with Wolfe line search.

The characteristics of the HS method are similar to the PRP method, but compared with PRP method, an important feature of the HS method is that no matter whether the line precision is exist, the conjugate relation *d*_*k*+1_^*T*^*y*_*k*_ = 0 is always formed.

The CD method is very similar with the FR method. Under the exact line search, *β*_*k*_^*CD*^ = *β*_*k*_^*FR*^; Under the inexact line search, an important feature of the CD algorithm is that as long as the parameter *σ* < 1 in strong Wolfe line search, the CD method would generate a descent search direction in each iteration, but its global convergence property is not good.

Under the exact line search, the LS method is equivalent to the PRP method.

The DY method can always generate a descent direction in each iteration with Wolfe line search, but the disadvantage of this method is the bad numerical experiment results.

In the paper, we learn and analyze the above methods, then proposed two hybrid nonlinear conjugate gradient method, namely, the hybrid method of DY and HS and the hybrid method of FR and PRP. We also have a research on both each methods.

## Hybrid conjugate gradient method of DY and HS

### Preliminaries of the new conjugate gradient method

To the hybrid conjugate gradient method of DY and HS, we promote the Wolfe line search in our paper. The standard Wolfe line search (5) is revised as the following:

If *d*_*k*_^T^*g*_*k*+1_ ≥ 0, then

8 $${\sigma_{1}} d_{k}^{\text{T}} g_{k} \le d_{k}^{\text{T}} g\left( {x_{k} + \alpha_{k} d_{k} } \right) \le - \sigma_{2} d_{k}^{\text{T}} g_{k}$$

where $$-{\sigma}_{2}d_{k}^{\text{T}} g_{k} \,{<} - \sigma_{2} d_{k}^{\text{T}} \left( {g_{k} - g_{k+1} } \right)$$

If *d*_*k*_^T^*g*_*k*+1_ < 0, then

9 $${\sigma_{1}} d_{k}^{\text{T}} {\text{g}}_{\text{k}} \le d_{k}^{\text{T}} {\text{g}}\left( {x_{\text{k}} + \alpha_{\text{k}} {\text{d}}_{\text{k}} } \right) \le - {\sigma_{2}} d_{k}^{\text{T}} \left( {{\text{g}}_{\text{k}} - {\text{g}}_{{{\text{k}} + 1}} } \right)$$

where $$- {\sigma}_{ 2} d_{k}^{\text{T}} \left( {g_{k} - g_{k + 1} } \right) < - \sigma_{ 2} d_{k}^{\text{T}} g_{k}.$$

The parameters *β*_*k*_ of the hybrid conjugate gradient method is formulized as

$$\beta _{k}^{{\left( 1 \right)}} = \left\{ {\begin{array}{*{20}ll} {a_{1} \beta _{k}^{{DY}} + a2\beta _{k}^{{HS}} } & \quad {if\,\left\| {gk} \right\|^{2} > \,\left| {g_{k}^{T} g_{{k - 1}} } \right|} \\ 0 & \quad{else} \\ \end{array} } \right.$$

i.e.

10 $$\beta _{k}^{{\left( 1 \right)}} = \left\{ {\begin{array}{*{20}ll} {\frac{{a_{1} \left\| {g_{k} } \right\|^{2} }}{{d_{{k - 1}}^{T} \left( {g_{k} - g_{{k - 1}} } \right)}} + \frac{{a_{2} g_{k}^{T} \left( {g_{k} - g_{{k - 1}} } \right)}}{{d_{{k - 1}}^{T} \left( {g_{k} - g_{{k - 1}} } \right)}}} & \quad {if\,\left\| {g_{k} } \right\|^{2} > \left| {g_{k}^{T} g_{{k - 1}} } \right|} \\ 0 &\quad {else} \\ \end{array} } \right.$$

where *a*_1_, *a*_2_ are nonnegative numbers and at least one are not zero, and that they are required to satisfy

11 $$0 < a_{1} + 2a_{2} < \frac{1}{{1 + \sigma_{2} }} < 1$$

A new Wolfe line search is proposed, which makes the hybrid conjugate gradient method keep the global convergence property and the descent property in this paper.

When *β*_*k*_ = 0, the new hybrid method will degenerate into the steepest descent method.

**Algorithm** (*The new hybrid method with the new Wolfe line search*)

Step 1: Choose an initial point *x*_1_ ∊ *R*^*n*^. Give the precision value *ɛ* > 0. Compute *g*_1_, if ‖*g*_1_‖ < *ɛ*, then stop, *x*_1_ is the optimal point; otherwise go to Step 2

Step 2: Set *d*_1_ = − *g*_1_. Let *k* = 1

Step 3: Set *x*_*k*+1_ = *x*_*k*_ + *α*_*k*_*d*_*k*_, *α*_*k*_ is defined by the new generalized Wolfe line search (4) (8) (9)

Step 4: Compute *g*_*k*+1_, if ‖*g*_*k*+1_‖ < *ɛ*, then stop; otherwise go to Step 5

Step 5: Set $k: = k + 1$; Set $d_{k} = - g_{k} + \beta_{k}^{1} d_{k - 1}$, *β*_*k*_^(1)^ is defined by the formula (10), then go to Step 3

### The descent property

**Assumption H**

H1: The objective function *f*(*x*) is a continuously differentiable function. The level set *L*_1_ = {*x* ∊ *R*^*n*^: *f*(*x*) ≤ *f*(*x*_1_)} at *x*_1_ is bounded (*x*_1_ is the initial point); namely, there exists a constant *a* > 0 such that

$$\left\| x \right\| \le a \quad {\text{for all}} \quad x \in L_{1}$$

H2: In any neighborhood *N* of *L*_1_, *f* is continuously differentiable, and its gradient *g*(*x*) is Lipschitz continuous with Lipschitz constant *L* > 0; i.e.,

$$\left\|{\text{g}}\left( x \right) - {\text{g}}\left( {\text{y}} \right)\right\| \le \left\|{\text{L}}x - y\right\|$$ for all *x*, *y* ∊ *N*

#### **Lemma 1**

*Suppose that the objective function satisfies Assumption H. Consider the method* (2), (3)*, where α*_*k*_*satisfies the new Wolfe line search* (4), (8), (9) *and β*_*k*_^*(1)*^*satisfies the formula* (10)*, then the following holds: *

$$g_{k}^{{\rm T}} d_{k} < 0 \quad {for \,\,all \,\,k}$$

*Proof* For *k* = 1, we have *g*_1_^T^*d*_1_ = *g*_1_^T^*g*_1_ = − ‖*g*_1_‖^2^ < 0 according to *d*_1_ = − *g*_1_.

For *k* > 1, suppose that *g*_*k*_^T^*d*_*k*_ < 0 holds at the k-th step, then we prove this inequality also holds at the k + 1-th step.

From ‖*g*_*k*_‖^2^ > |*g*_*k*_^T^*g*_*k*−1_| and (11), we have that

$$\begin{aligned} g_{k + 1}^{\rm T} d_{k + 1} &= - \| {g_{k + 1} }\|^{2} + \beta_{k + 1}^{(1)} g_{k + 1}^{\rm T} d_{k} \\ &= - \| {g_{k + 1} }\|^{2} + \left [\frac{{a_{1} \| {g_{k + 1} } \|^{2} }}{{d_{k}^{\rm T} (g_{k + 1} - g_{k} )}} + \frac{{a_{2} g_{k + 1}^{\rm T} (g_{k + 1} - g_{k} )}}{{d_{k}^{\rm T} (g_{k + 1} - g_{k} )}} \right ] \cdot g_{k + 1}^{\rm T} d_{k} \\ & \le - \| {g_{k + 1} } \|^{2} + \frac{{(a_{1} + a_{2} ) \| {g_{k + 1} }\|^{2} - a_{2} g_{k + 1}^{\rm T} g_{k} }}{{d_{k}^{\rm T} (g_{k + 1} - g_{k} )}}( - \sigma_{2} d_{k}^{\rm T} (g_{k} - g_{k + 1} )) \\ & \le - \| {g_{k + 1} } \|^{2} + \frac{{(a_{1} + 2a_{2} ) \| {g_{k + 1} } \|^{2} }}{{d_{k}^{\rm T} (g_{k + 1} - g_{k} )}} \cdot ( - \sigma_{2} d_{k}^{\rm T} (g_{k} - g_{k + 1} )) \\ &= [(a_{1} + 2a_{2} )\sigma_{2} - 1] \| {g_{k + 1} }\|^{2} \end{aligned}$$

Then, by $0 < a_{1} + 2a_{2} < \frac{1}{{1 + \sigma_{2} }} < 1$ and 0 ≤ *σ*_2_ < 1, we have *g*_*k*+1_^T^*d*_*k*+1_ < 0.

Therefore, according to the mathematical induction, Lemma 1 is proved, which implies that the new hybrid method has the descent property.

### Global convergence

#### **Lemma 2**

*Assume that (H) hold, Consider the method* (2), (3), *whered*_*k*_* is a descent direction and α*_*k*_*satisfies the new Wolfe line search* (4), (8), (9), *β*_*k*_^(1)^*satisfies the formula *(10). *Then we have that*$\sum\nolimits_{k \ge 1} {\frac{{(g_{k}^{\rm T} d_{k} )^{2} }}{{\left\| {d_{k} } \right\|^{2} }}} < \infty$

*Proof* By Lemma 1, we have *g*_*k*_^T^*d*_*k*_ < 0, so the sequence {*f*(*x*_*k*_)} is bounded and has monotone descending property, which implies that {*f*(*x*_*k*_)} is a convergent sequence. From (8), (9), we have

12 $$0 < \left( {{\sigma}_{1} - 1} \right){\text{g}}_{\text{k}}^{\text{T}} {\text{d}}_{\text{k}} \le {\text{d}}_{\text{k}}^{\text{T}} \left( {{\text{g}}_{{{\text{k+ 1}}}} - {\text{g}}_{\text{k}} } \right) \le - (1 + {\sigma}_{2} ){\text{g}}_{\text{k}}^{\text{T}} {\text{d}}_{\text{k}}$$

From the Assumption H2, we have

13 $$d_{k}^{\rm T} (g_{k + 1} - g_{k} ) \le L\alpha_{k} \left\| {d_{k} } \right\|^{2}$$

From (12), (13), we have

14 $$\alpha_{k} \ge \frac{{g_{k}^{\rm T} d_{k} (\sigma_{1} - 1)}}{{L\left\| {d_{k} } \right\|^{2} }}$$

From (8), (14), we have

$$f_{k} - f_{k + 1} \ge - \mu \alpha_{k} g_{k}^{\rm T} d_{k} \ge \frac{{ - \mu (g_{k}^{\rm T} d_{k} )^{2} (\sigma_{1} - 1)}}{{L\left\| {d_{k} } \right\|^{2} }} = \frac{{\mu (1 - \sigma_{1} )}}{L} \cdot \frac{{(g_{k}^{\rm T} d_{k} )^{2} }}{{\left\| {d_{k} } \right\|^{2} }}$$

By summing this formula, we have

$$\sum\limits_{k \ge 1} {(f_{k} - f_{k + 1} ) = } \,f_{1} - \mathop {\lim }\limits_{k \to \infty } f_{k} \ge \sum\limits_{k \ge 1} {\frac{{\mu (1 - \sigma_{1} )}}{L} \cdot \frac{{(g_{k}^{\rm T} d_{k} )^{2} }}{{\left\| {d_{k} } \right\|^{2} }}}$$

Then

$$\sum\limits_{k \ge 1} {\frac{{(g_{k}^{\rm T} d_{k} )^{2} }}{{\left\| {d_{k} } \right\|^{2} }}} < \infty$$

The proof is completed.

#### **Theorem 3**

*Suppose that Assumption H1 and H2 are satisfied. Consider the method* (2), (3), *where α*_*k*_*satisfies the new Wolfe line search* (4), (8), (9)*, β*_*k*_^*(1)*^*satisfies the formula* (10). *Then the following holds: * 

$$\mathop {\lim }\limits_{{k \to \infty }} \inf \left\| {g_{k} } \right\| = 0$$

*Proof* If lim _*k*→∞_ inf ‖*g*_*k*_‖ = 0 is not true, there exists a constant *c* > 0 such that

15 $$\left\| {g_{k} } \right\|^{2} > c, \quad {\text{for}}\,{\text{all}}\,{\text{k}}$$

Therefore, from $d_{k} = { - }g_{k} + \beta_{k}^{\left( 1 \right)} d_{{k{ - }1}}$, multiplying with *g*_*k*_ on the both sides, we have $g_{k}^{\rm T} d_{k} = - \left\| {g_{k}^{{}} } \right\|^{2} + \beta_{k}^{(1)} g_{k}^{\text{T}} d_{k - 1}$. Thus (8) and (9) yield

$$- \sigma _{2} < \frac{{g_{k}^{{\text{T}}} d_{{k - 1}} }}{{g_{{k - 1}}^{{\text{T}}} d_{{k - 1}} }} < \sigma _{1}$$

Then

16 $$\frac{{\sigma _{1} }}{{\sigma _{{1 - 1}} }} < \frac{{g_{k}^{{\text{T}}} d_{{k - 1}} }}{{d_{{k - 1}}^{{\text{T}}} \left( {g_{k} - g_{{k - 1}} } \right)}} < \frac{{\sigma _{2} }}{{\sigma _{2} + 1}}$$

On the other hand, from ‖*g*_*k*_‖^2^ > |*g*_*k*_^T^*g*_*k*−1_|, we have

$$\begin{aligned} \frac{{ - g_{k}^{\rm T} d_{k} }}{{\left\| {g_{k} } \right\|^{2} }} &= 1 - \frac{{\beta_{k}^{(1)} g_{k}^{\rm T} d_{k - 1} }}{{\left\| {g_{k} } \right\|^{2} }} \\ &= 1 - \left[ {\frac{{a_{1} \left\| {g_{k} } \right\|^{2} }}{{d_{k - 1}^{\rm T} (g_{k} - g_{k - 1} )}} + \frac{{a_{2} g_{k}^{\rm T} (g_{k} - g_{k - 1} )}}{{d_{k - 1}^{\rm T} (g_{k} - g_{k - 1} )}}} \right] \cdot \frac{{g_{k}^{\rm T} d_{k - 1} }}{{\left\| {g_{k} } \right\|^{2} }} \\ \end{aligned}$$

If *g*_*k*_^T^*d*_*k*−1_ ≥ 0, then

$$\begin{aligned} \frac{{ - g_{k}^{\rm T} d_{k} }}{{\left\| {g_{k} } \right\|^{2} }} &\ge 1 - \frac{{(a_{1} + 2a_{2} )\left\| {g_{k} } \right\|^{2} }}{{\left\| {g_{k} } \right\|^{2} }} \cdot \frac{{g_{k}^{\rm T} d_{k - 1} }}{{d_{k - 1}^{\rm T} (g_{k} - g_{k - 1} )}} \hfill \\&= 1 - (a_{1} + 2a_{2} ) \cdot \frac{{g_{k}^{\rm T} d_{k - 1} }}{{d_{k - 1}^{\rm T} (g_{k} - g_{k - 1} )}} \hfill \\ \end{aligned}$$

If *g*_*k*_^T^*d*_*k*−1_ < 0, then

$$\begin{aligned} \frac{{ - g_{k}^{\rm T} d_{k} }}{{\left\| {g_{k} } \right\|^{2} }} &\le 1 - \frac{{(a_{1} + 2a_{2} )\left\| {g_{k} } \right\|^{2} }}{{\left\| {g_{k} } \right\|^{2} }} \cdot \frac{{g_{k}^{\rm T} d_{k - 1} }}{{d_{k - 1}^{\rm T} (g_{k} - g_{k - 1} )}} \\ &= 1 - (a_{1} + 2a_{2} ) \cdot \frac{{g_{k}^{\rm T} d_{k - 1} }}{{d_{k - 1}^{\rm T} (g_{k} - g_{k - 1} )}} \hfill \\ \end{aligned}$$

Therefore, by (16), we have

17 $$1 - (a_{1} + 2a_{2} )\frac{{\sigma_{2} }}{{\sigma_{2} + 1}} \le \frac{{ - g_{k}^{\rm T} d_{k} }}{{\left\| {g_{k} } \right\|^{2} }} \le 1 - (a_{1} + 2a_{2} ) \cdot \frac{{\sigma_{1} }}{{\sigma_{1} - 1}}$$

And, by squaring both sides of $d_{k} + g_{k} = \beta_{k}^{\left( 1 \right)} d_{k - 1}$, we have

$$\left\| {d_{k} } \right\|^{2} = - \left\| {g_{k} } \right\|^{2} - 2g_{k}^{\rm T} d_{k} + (\beta_{k}^{(1)} )^{2} \left\| {d_{k - 1} } \right\|^{2}$$

Then by multiplying with (*g*_*k*_^T^*d*_*k*_)^2^ on the both sides, we have

$$\begin{aligned} \frac{{\left\| {d_{k} } \right\|^{2} }}{{(g_{k}^{\rm T} d_{k} )^{2} }} &= \frac{{(\beta_{k}^{(1)} )^{2} \left\| {d_{k - 1} } \right\|^{2} }}{{(g_{k}^{\rm T} d_{k} )^{2} }} - \frac{2}{{g_{k}^{\rm T} d_{k} }} - \frac{{\left\| {g_{k} } \right\|^{2} }}{{(g_{k}^{\rm T} d_{k} )^{2} }} \hfill \\ {\kern 1pt} {\kern 1pt} {\kern 1pt} {\kern 1pt} {\kern 1pt} {\kern 1pt} {\kern 1pt} {\kern 1pt} {\kern 1pt} {\kern 1pt} {\kern 1pt} {\kern 1pt} {\kern 1pt} {\kern 1pt} {\kern 1pt} {\kern 1pt} {\kern 1pt} {\kern 1pt} {\kern 1pt} {\kern 1pt} {\kern 1pt} {\kern 1pt} {\kern 1pt} {\kern 1pt} {\kern 1pt} {\kern 1pt} {\kern 1pt} {\kern 1pt} {\kern 1pt} {\kern 1pt} {\kern 1pt} {\kern 1pt} {\kern 1pt} {\kern 1pt} {\kern 1pt} {\kern 1pt} {\kern 1pt} {\kern 1pt} {\kern 1pt} {\kern 1pt} {\kern 1pt} &= \frac{{(\beta_{k}^{(1)} )^{2} \left\| {d_{k - 1} } \right\|^{2} }}{{(g_{k}^{\rm T} d_{k} )^{2} }} - \left(\frac{1}{{\left\| {g_{k} } \right\|}} + \frac{{\left\| {g_{k} } \right\|}}{{g_{k}^{\rm T} d_{k} }}\right)^{2} + \frac{1}{{\left\| {g_{k} } \right\|^{2} }} \hfill \\ {\kern 1pt} {\kern 1pt} {\kern 1pt} {\kern 1pt} {\kern 1pt} {\kern 1pt} {\kern 1pt} {\kern 1pt} {\kern 1pt} {\kern 1pt} {\kern 1pt} {\kern 1pt} {\kern 1pt} {\kern 1pt} {\kern 1pt} {\kern 1pt} {\kern 1pt} {\kern 1pt} {\kern 1pt} {\kern 1pt} {\kern 1pt} {\kern 1pt} {\kern 1pt} {\kern 1pt} {\kern 1pt} {\kern 1pt} {\kern 1pt} {\kern 1pt} {\kern 1pt} {\kern 1pt} {\kern 1pt} {\kern 1pt} {\kern 1pt} {\kern 1pt} {\kern 1pt} {\kern 1pt} {\kern 1pt} {\kern 1pt} {\kern 1pt} {\kern 1pt} &\le \frac{{(\beta_{k}^{(1)} )^{2} \left\| {d_{k - 1} } \right\|^{2} }}{{(g_{k}^{\rm T} d_{k} )^{2} }} + \frac{1}{{\left\| {g_{k} } \right\|^{2} }} \hfill \\ \end{aligned}$$

However, because of

$$(\beta_{k}^{(1)} )^{2} = \left[\frac{{a_{1} \left\| {g_{k} } \right\|^{2} }}{{d_{k - 1}^{\rm T} (g_{k} - g_{k - 1} )}}{ + }\frac{{a_{2} g_{k}^{\rm T} (g_{k} - g_{k - 1} )}}{{d_{k - 1}^{\rm T} (g_{k} - g_{k - 1} )}}\right]^{2} \le \frac{{(a_{1} + 2a_{2} )^{2} \left\| {g_{k} } \right\|^{4} }}{{(d_{k - 1}^{\rm T} (g_{k} - g_{k - 1} ))^{2} }},$$

and thus from (17), we have

$$\begin{aligned} \frac{{\left\| {d_{k} } \right\|^{2} }}{{(g_{k}^{\rm T} d_{k} )^{2} }} &\le \frac{{(a_{1} + 2a_{2} )^{2} \left\| {g_{k} } \right\|^{4} }}{{(d_{k}^{\rm T} g_{k} )^{2} }} \cdot \frac{{\left\| {d_{k - 1} } \right\|^{2} }}{{(d_{k - 1}^{\rm T} (g_{k} - g_{k - 1} ))^{2} }} + \frac{1}{{\left\| {g_{k} } \right\|^{2} }} \hfill \\ {\kern 1pt} {\kern 1pt} {\kern 1pt} {\kern 1pt} {\kern 1pt} {\kern 1pt} {\kern 1pt} {\kern 1pt} {\kern 1pt} {\kern 1pt} {\kern 1pt} {\kern 1pt} {\kern 1pt} {\kern 1pt} {\kern 1pt} {\kern 1pt} {\kern 1pt} {\kern 1pt} {\kern 1pt} {\kern 1pt} {\kern 1pt} {\kern 1pt} {\kern 1pt} {\kern 1pt} {\kern 1pt} {\kern 1pt} {\kern 1pt} {\kern 1pt} {\kern 1pt} {\kern 1pt} {\kern 1pt} {\kern 1pt} {\kern 1pt} {\kern 1pt} {\kern 1pt} {\kern 1pt} {\kern 1pt} {\kern 1pt} {\kern 1pt} {\kern 1pt} {\kern 1pt} {\kern 1pt} &\le \frac{{(a_{1} + 2a_{2} )^{2} }}{{[1 - (a_{1} + 2a_{2} )\frac{{\sigma_{2} }}{{\sigma_{2} + 1}}]^{2} }} \cdot \frac{{\left\| {d_{k - 1} } \right\|^{2} }}{{(d_{k - 1}^{\rm T} (g_{k} - g_{k - 1} ))^{2} }} + \frac{1}{{\left\| {g_{k} } \right\|^{2} }} \hfill \\ {\kern 1pt} {\kern 1pt} {\kern 1pt} {\kern 1pt} {\kern 1pt} {\kern 1pt} {\kern 1pt} {\kern 1pt} {\kern 1pt} {\kern 1pt} {\kern 1pt} {\kern 1pt} {\kern 1pt} {\kern 1pt} {\kern 1pt} {\kern 1pt} {\kern 1pt} {\kern 1pt} {\kern 1pt} {\kern 1pt} {\kern 1pt} {\kern 1pt} {\kern 1pt} {\kern 1pt} {\kern 1pt} {\kern 1pt} {\kern 1pt} {\kern 1pt} {\kern 1pt} {\kern 1pt} {\kern 1pt} {\kern 1pt} {\kern 1pt} {\kern 1pt} {\kern 1pt} {\kern 1pt} {\kern 1pt} {\kern 1pt} {\kern 1pt} {\kern 1pt} {\kern 1pt} {\kern 1pt} &\mathop = \limits^{\Delta } m \cdot \frac{{\left\| {d_{k - 1} } \right\|^{2} }}{{(d_{k - 1}^{\rm T} (g_{k} - g_{k - 1} ))^{2} }} + \frac{1}{{\left\| {g_{k} } \right\|^{2} }}(m > 0) \\ \end{aligned}$$

By using the recurrence method on the left-hand side of the above inequality, there exists a constant *T* > 0 such that

$$\frac{{\left\| {d_{k} } \right\|^{2} }}{{(g_{k}^{\rm T} d_{k} )^{2} }} \le \sum\limits_{i = 1}^{k} {\frac{{m^{k - i} }}{{\left\| {g_{k} } \right\|^{2} }}} \le \frac{1}{c}\sum\limits_{i = 1}^{k} {m^{k - i} \le T}$$

By summing the above inequality, we have $\sum\nolimits_{k \ge 1} {\frac{{(g_{k}^{\rm T} d_{k} )^{2} }}{{\left\| {d_{k} } \right\|^{2} }}} \ge \sum\nolimits_{k \ge 1} {1/T = + \infty }$, which contradicts Lemma 2. So the proof is complete.

## Hybrid conjugate gradient method of FR and PRP

As the hybrid conjugate gradient method of DY and HS, we promote the Wolfe line search as well in our paper. The standard Wolfe line search (5) is revised as the following:

If $$- \left\| {g_{k} } \right\|^{2} \le d_{k}^{T} g_{k}^{T}$$ then

18 $$\sigma_{1} d_{k}^{T} g_{k}^{T} \le d_{k}^{T} g_{k + 1}^{T} \le - \sigma_{2} d_{k}^{T} g_{k}^{T}$$

If $$- \left\| {g_{k} } \right\|^{2} > d_{k}^{T} g_{k}^{T}$$ then

19 $$- \sigma_{1} \left\| {g_{k} } \right\|^{2} \le d_{k}^{T} g_{k + 1}^{T} \le \sigma_{2} \left\| {g_{k} } \right\|^{2}$$

The parameters *β*_*k*_ of the hybrid conjugate gradient method of FR and PRP is formulized as

20 $$\beta_{\text{k}}^{(2)} = \left\{ \begin{array}{*{20}ll} a_{1} \beta_{\text{k}}^{\text{FR}} + a_{2} \beta_{\text{k}}^{\text{PRP}} & \quad if\, \left\|{\text{g}}_{\text{k}}\right\|^{2} > \left| {{\text{g}}_{\text{k}}^{\text{T}} {\text{g}}_{{{\text{k}} - 1}} } \right| \\ 0&\quad {\text{else }} \\ \end{array} \right.$$

where *a*_1_, *a*_2_ are nonnegative parameters and also at least one is not zero, and they are required to satisfy

21 $$0 < a_{1} + 2a_{2} < \frac{1}{{1 + \sigma_{2} }} < 1$$

The new Wolfe line search also can make the hybrid conjugate gradient method of FR and PRP keep the global convergence property and the descent property in this paper.

Both the properties can be proofed by the same process as the hybrid method of DY and HS.

## Numerical experiments

In this section, we report some preliminary numerical experiments. We chose 15 test problems (problems 21–35) with the dimension n = 10,000 and initial points from the literature (More et al. 1981) to implement the two hybrid methods with the new line search with a portable computer. The stop criterion is ‖*g*_*k*_‖ ≤ 10^−6^ and we set the parameters as *a*_1_ = 0.2, *a*_2_ = 0.2, *σ*_1_ = *σ*_2_ = 0.6 and *μ* = 0.4. Four conjugate gradient algorithms (DY, Hybrid conjugate gradient method of DY and HS, PRP, Hybrid conjugate gradient method of FR and PRP) are compared in numerical performance and the numerical results are given in Table 1.

**Table 1:** Number of iterations and number of functional evaluations

p	n	DY	DY and HS	PRP	FR and PRP
21	10^4	72/222	70/212	58/179	50/189
22	10^4	74/532	70/520	63/429	58/409
23	10^4	62/241	60/241	41/198	40/176
24	10^4	89/304	89/300	82/297	82/302
25	10^4	51/153	44/143	46/127	44/107
26	10^4	55/202	56/202	52/198	46/188
27	10^4	45/246	46/238	52/239	42/209
28	10^4	91/425	84/415	84/337	80/312
29	10^4	58/245	55/240	45/211	39/183
30	10^4	53/308	48/298	43/281	43/277
31	10^4	74/268	74/258	76/281	70/265
32	10^4	77/457	74/447	87/421	82/425
33	10^4	48/172	38/152	37/136	33/126
34	10^4	82/396	80/383	58/385	56/354
35	10^4	76/342	74/322	72/276	68/256
CPU	–	355 s	312 s	252 s	231 s

In Table 1, CPU denotes the CPU time (seconds) for solving all the 15 test problems. A pair numbers means the number of iterations and the number of functional evaluations. It can be seen from Table 1 that two hybrid methods with the new Wolfe line search is effective for solving some large scale problems. In particular, the Hybrid conjugate gradient method of FR and PRP seems to be the best one among the four algorithms because it uses the least number of iterations and functional evaluations when the algorithms reach the same precision.

## Conclusion

In this paper, we have proposed two hybrid conjugate gradient methods, respectively are the hybrid method of DY and HS, the hybrid method of FR and PRP. Moreover, we have proposed the corresponding new Wolfe line search, which make the corresponding hybrid method keep the global convergence property and the descent property.

Dai and Yuan have proposed a family of the three-term conjugate gradient method:

$$\beta_{k} = \frac{{(1 - \lambda_{k} )\left\| {g_{k} } \right\|^{2} + \lambda_{k} g_{k}^{\rm T} (g_{k} - g_{k - 1} )}}{{(1 - \mu_{k} - \omega_{k} )\left\| {g_{k - 1} } \right\|^{2} + \mu_{k} d_{k - 1}^{\rm T} (g_{k} - g_{k - 1} ) - \omega_{k} d_{k - 1}^{\rm T} g_{k - 1} }}$$

where *λ*_*k*_ ∊ [0, 1], *μ*_*k*_ ∊ [0, 1], *ω*_*k*_ ∊ [0, 1−*μ*_*k*_].

Let *μ*_*k*_ = 1, *ω*_*k*_ = 0, we have

22 $$\beta_{k} = \frac{{(1 - \lambda_{k} )\left\| {g_{k} } \right\|^{2} + \lambda_{k} g_{k}^{\rm T} (g_{k} - g_{k - 1} )}}{{d_{k - 1}^{\rm T} (g_{k} - g_{k - 1} )}}$$

Let *μ*_*k*_ = *ω*_*k*_ = 0, then we have

23 $$\beta_{k} = \frac{{(1 - \lambda_{k} )\left\| {g_{k} } \right\|^{2} + \lambda_{k} g_{k}^{\rm T} (g_{k} - g_{k - 1} )}}{{\left\| {g_{k - 1} } \right\|^{2} }}$$

In our paper, *β*_*k*_ can be rewritten as

$$\beta_{k}^{(1)} = \frac{{a_{1} \left\| {g_{k} } \right\|^{2} + a_{2} g_{k}^{\rm T} (g_{k} - g_{k - 1} )}}{{d_{k - 1}^{\rm T} (g_{k} - g_{k - 1} )}}$$

$$\beta_{k}^{(2)} = \frac{{a_{1} \left\| {g_{k} } \right\|^{2} + a_{2} g_{k}^{\rm T} (g_{k} - g_{k - 1} )}}{{\left\| {g_{k - 1} } \right\|^{2} }}$$

The difference between (22), (23) and *β*_*k*_^(1)^, *β*_*k*_^(2)^ defined in the paper is the numerator ‖*g*_*k*_‖^2^ and *g*_*k*_^T^(*g*_*k*_ − *g*_*k*−1_) are convex combination of the formula (22), (23), however, in the formula *β*_*k*_^(1)^, *β*_*k*_^(2)^, *a*_1_ + *a*_2_ ≠ 1, which have weakened the condition.
